# Evaluating 24-h Urine Aldosterone Levels as a Practical Diagnostic Tool for Primary Aldosteronism

**DOI:** 10.3390/jcm15124503

**Published:** 2026-06-10

**Authors:** Rıza Gökhan Baykal, Berna Evranos Öğmen, Sevilay Sezer, Sevgül Fakı, Fatma Dilek Dellal Kahramanca, Cevdet Aydın, Oya Topaloğlu, Reyhan Ersoy, Bekir Çakır

**Affiliations:** 1 Department of Endocrinology and Metabolism, Ankara Bilkent City Hospital, Ankara 06800, Turkeydrdellal@yahoo.com (F.D.D.K.); 2 Department of Endocrinology and Metabolism, Faculty of Medicine, Ankara Yildirim Beyazit University, Ankara 06800, Turkey; evranosberna@gmail.com (B.E.Ö.); oyasude@gmail.com (O.T.); reyhanersoy@yahoo.com.tr (R.E.);; 3 Department of Medical Biochemistry, Ankara Bilkent City Hospital, Ankara 06800, Turkey; sevilaysezer@gmail.com

**Keywords:** primary aldosteronism, urinary aldosterone, suppressed renin, diagnosis, confirmatory tests

## Abstract

**Background/Objectives**: This study aimed to evaluate the diagnostic utility of 24 h urinary aldosterone (uAldo) measurements in patients with suspected primary aldosteronism (PA) and to explore its potential utility in guiding individualized clinical decisions. **Methods**: We examined 40 patients with suspected PA who underwent screening, confirmatory testing and 24 h uAldo assessment at a tertiary endocrinology center. Confirmatory tests were performed per standardized protocols. Patients were classified into three groups based on uAldo levels derived from receiver operating characteristic (ROC) analysis: Group 1 (<8.7 μg/24 h), Group 2 (8.7–13.6 μg/24 h), and Group 3 (>13.6 μg/24 h). Diagnostic performance was evaluated using sensitivity, specificity, and area under the curve (AUC). **Results**: uAldo levels were significantly higher in patients with positive confirmatory tests (16.3 μg/24 h vs. 5.5 μg/24 h, *p* < 0.001). A cut-off of >8.7 μg/24 h showed 89% sensitivity and 91% specificity (AUC: 0.95), while >13.6 μg/24 h yielded 91% sensitivity and 86% specificity (AUC: 0.97). Patients in Group 2 were managed solely with medical therapy, with no need for invasive procedures. Group 3 patients had higher blood pressure, higher uAldo levels, and more frequent hypokalemia. Adrenal venous sampling and surgery were more common in Group 3, with histological PA confirmation in all operated patients. **Conclusions**: The 24 h uAldo measurement demonstrated promising diagnostic performance in this cohort. The proposed cut-offs may help support patient stratification and clinical decision-making in patients with suspected PA. However, because confirmatory testing served as the reference standard, these findings should be considered exploratory and require external validation in larger multicenter studies before incorporation into routine diagnostic algorithms.

## 1. Introduction

Primary aldosteronism (PA) is a common and underdiagnosed cause of secondary hypertension, with an increasing prevalence of 5–20%, depending on the study population [1,2,3,4,5,6]. Emerging evidence further suggests that PA may represent a broader pathophysiological continuum extending beyond classical hypokalemic phenotypes [7]. It is underrecognized due to limitations in current screening and diagnostic approaches [2,6,8]. Although a stepwise diagnostic strategy involving laboratory evaluations and confirmatory testing is the standard approach, it often presents practical and clinical challenges. The aldosterone-renin ratio (ARR) is widely used for initial screening of PA; however, its diagnostic reliability can be affected by various physiological and pharmacological factors, resulting in false positives or negatives [9,10]. Therefore, confirmatory testing is essential to demonstrate autonomous aldosterone excess independent of renin activity [2,4,11,12]. Recent European guidelines continue to recommend confirmatory testing in most patients with suspected PA, although important practical limitations, assay variability, and challenges in standardization remain clinically relevant [13]. However, plasma aldosterone secretion has been shown to have pulsatile and fluctuating patterns and also to exhibit considerable intra-individual variability in patients with PA [14,15,16]. Furthermore, determination of plasma aldosterone is methodologically challenging, and there are factors such as preanalytical conditions and assay variability that may influence results [17,18,19]. Suppressed plasma renin activity (PRA) is a key indicator of autonomous aldosterone production, but its levels can be affected by serum potassium, dietary sodium intake, medications, and patient posture [20,21]. In contrast, 24 h urinary aldosterone (uAldo) excretion minimizes the effects of diurnal fluctuations and episodic secretion, providing a more accurate estimate of aldosterone production over time [22,23]. Furthermore, patients with PA experience higher rates of cardiovascular events and target organ damage compared to those with essential hypertension [24,25]. Complications may also arise during confirmatory testing, especially in patients with significant comorbidities or severe hypertension [26,27]. Recent studies have further supported the diagnostic performance of uAldo measurements and proposed practical cut-off values, reinforcing its utility. However, these protocols often require specific preconditions such as sodium loading or potassium repletion. This approach may reduce the need for confirmatory testing and mitigate the risks associated with invasive testing [28,29,30]. Unlike classical protocols that require formal sodium loading and measurement of 24 h urinary sodium to ensure adequate intake, our study evaluated the diagnostic utility of uAldo measurements obtained under a normal-sodium diet, using suppressed renin activity as a physiological criterion. By performing ROC analysis on this “real-world” data collection approach, we aimed to derive stratification cut-offs that can guide clinical decision-making. This practical methodology may represent a simpler and more accessible confirmatory step for diagnosing PA, especially in resource-limited or outpatient settings. Given the increasing recognition and clinical burden of PA, there is a growing need for practical and accurate diagnostic alternatives. The aim of this study was to evaluate the diagnostic value of 24 h urinary aldosterone levels in the context of suppressed renin activity, and to compare its performance with classical confirmatory tests.

## 2. Materials and Methods

### 2.1. Study Design

This was a single-center observational cohort study using routinely collected clinical data, analyzed retrospectively. The study was conducted at the Endocrinology Department of Ankara Bilkent City Hospital between January 2023 and May 2025. Patients admitted with suspected primary aldosteronism underwent standardized diagnostic evaluations, including biochemical tests and confirmatory testing, during hospitalization. Patients were evaluated for primary aldosteronism based on current clinical indications, including resistant hypertension, spontaneous or diuretic-induced hypokalemia, adrenal incidentaloma/adrenal adenoma, young-onset hypertension, suppressed renin activity with elevated aldosterone-renin ratio, or clinical suspicion of secondary hypertension. Medical history, demographic data, clinical findings, laboratory values such as liver and kidney function, serum electrolytes, plasma aldosterone concentration (PAC), PRA, 24 h urine aldosterone levels, imaging reports, confirmatory tests, adrenal venous sampling (AVS), and pathology reports for surgically treated patients were recorded. Following initial evaluation, patients were clinically monitored to assess treatment outcomes, including antihypertensive management, AVS, or surgical intervention when applicable. Patients with severe comorbidities (e.g., advanced heart failure, chronic kidney disease stage ≥4, or inability to undergo confirmatory testing) were excluded from the study. Based on confirmatory test results, patients were unequivocally classified as PA (at least one positive confirmatory test) or non-PA (negative confirmatory testing). In total, 18 patients met the biochemical criteria for PA, whereas 22 patients had negative confirmatory tests and served as the comparison group. There were no indeterminate or discordant cases.

### 2.2. Diagnostic Work-Up and Confirmatory Testing

**Figure 1:**
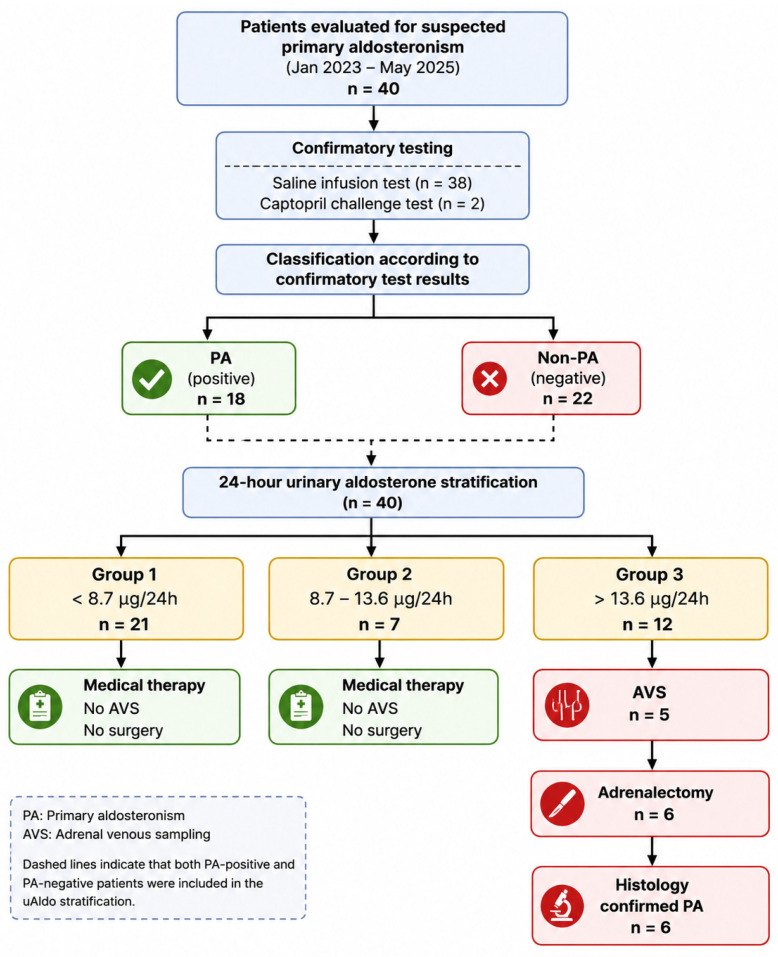
Study flowchart illustrating patient selection, confirmatory testing, classification according to primary aldosteronism status, urinary aldosterone-based stratification, and subsequent clinical management pathways including medical treatment, adrenal venous sampling, and adrenalectomy.

Confirmatory tests were performed at our center. Antihypertensive drugs affecting the Renin–Angiotensin–Aldosterone system were discontinued at least two weeks before the evaluation, and blood pressure control was provided with non-dihydropyridine calcium channel blockers or α-adrenergic antagonists. Patients with an elevated ARR (ARR ≥ 20 ng/dL per ng/mL/h) and suppressed renin were considered for confirmatory testing. Based on confirmatory test results, patients were unequivocally classified as PA (at least one positive confirmatory test) or non-PA (negative confirmatory testing). At least one positive test was confirmed: PAC > 11 ng/dL and inhibition ratio < 30% after the captopril challenge test, or PAC > 10 ng/dL in a sitting position after the saline infusion test [10]. The saline infusion test cut-off used in this study reflected the institutional diagnostic protocol routinely applied at our center throughout the study period. After correction of hypokalemia and adequate blood pressure control, 24 h urine samples were collected before confirmatory testing. All patients were placed on a normal sodium diet for three days before both urine collection and confirmatory procedures. Subsequently, each patient underwent a saline infusion test (*n* = 38) or captopril challenge test (*n* = 2) based on their clinical status and contraindications for the tests. Final clinical decisions regarding further testing, medical treatment, AVS, or surgical management were made in a multidisciplinary team meeting including endocrinologists, radiologists, and surgeons. Based on receiver operating characteristic (ROC) curve analysis, patients were categorized into three groups according to their uAldo levels: Group 1 (<8.7 μg/24 h, low uAldo), Group 2 (8.7–13.6 μg/24 h, intermediate uAldo), and Group 3 (>13.6 μg/24 h, high uAldo). The overall study design, patient classification, urinary aldosterone-based stratification, and subsequent management pathways are summarized in Figure 1.

### 2.3. Hormonal and Laboratory Measurements

PAC and PRA were measured after 1 h in the upright position using radioimmunoassay (RIA) kits (DiaSource, Louvain-la-Neuve, Belgium) at the Department of Biochemistry, Ankara Bilkent City Hospital. The limits of quantification (LOQ) were 25 pg/mL for PAC and 0.28 ng/mL/h for PRA. Intra- and inter-assay coefficients of variation were 3.8–13.7% and 6.2–18.6% for PAC and 2.7–3.9% and 5.0–5.1% for PRA, respectively. Urinary aldosterone was measured at Istanbul Acibadem Labmed Clinical Laboratory using a fully automated chemiluminescence immunoassay (LIAISON^®^, DiaSorin, Stillwater, MN, USA). The LOQ was 2.8 ng/dL, with intra- and inter-assay CVs of 2.3–5.6% and 5.4–7.9%, respectively. Forty patients met the inclusion criteria and completed both 24 h urine collection and confirmatory testing.

### 2.4. Statistical Analysis

Statistical analysis was performed using SPSS version 30.0 (IBM Corp., Armonk, NY, USA). The Kolmogorov–Smirnov test was applied to assess the normality of the distribution of continuous variables. Normally distributed variables were expressed as mean ± standard deviation, whereas non-normally distributed variables were expressed as median (Q1–Q3). Student’s *t*-test or Mann–Whitney U test was used for comparisons between two independent groups, as appropriate according to data distribution. Categorical variables were presented as counts and percentages [*n* (%)], and comparisons between groups were performed using the chi-square (χ^2^) test. The area under the ROC analysis was used to determine the diagnostic performance of the 24 h urinary aldosterone level. The lower cut-off value of >8.7 μg/24 h was determined based on ROC analysis performed on the full study cohort (*n* = 40), comparing patients with positive versus negative confirmatory test results. In contrast, the higher threshold of >13.6 μg/24 h was derived from a secondary ROC analysis restricted to patients with a positive confirmatory test (*n* = 18), where individuals were further stratified based on post-test plasma aldosterone levels into borderline (5–10 ng/dL) and clearly positive (>10 ng/dL) subgroups. The area under the curve (AUC) was calculated to evaluate the overall discriminatory ability. The optimal cut-off value for uAldo was determined using the Youden index, which maximizes the sum of sensitivity and specificity. Since this was a retrospective observational study, no formal a priori sample size calculation was performed. All eligible patients evaluated during the predefined study period were included. Post hoc power analysis was conducted using G*Power 3.1. Based on the observed effect size for urinary aldosterone levels between patients with positive and negative confirmatory tests (Cohen’s d = 2.13), the power of the study was calculated to be 1.00 (100%) at a significance level of α = 0.05, indicating excellent statistical power.

## 3. Results

**Figure 2:**
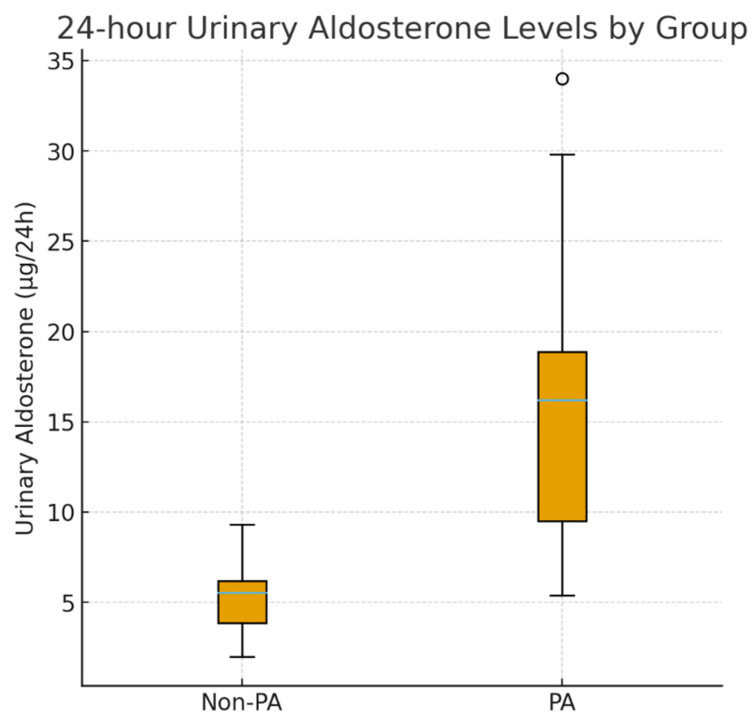
Box-and-whisker plot showing the distribution of 24 h urinary aldosterone (uAldo) levels in patients with primary aldosteronism (PA, *n* = 18) and those without PA (non-PA, *n* = 22). The boxes represent the interquartile range (IQR), the horizontal line within each box indicates the median, and the whiskers denote the minimum and maximum values within 1.5 × IQR. A significantly higher uAldo level was observed in the PA group compared with the non-PA group (*p* < 0.001).

A total of 40 patients were included in the study, including 18 patients with positive confirmatory tests for PA and 22 with negative confirmatory tests. Baseline demographic and clinical characteristics are summarized in Table 1. There were no indeterminate or discordant cases, and no patients with initially positive confirmatory results were subsequently reclassified as non-PA after further evaluation. Median uAldo excretion was significantly higher in patients with a positive confirmatory test [16.3 μg/24 h (IQR: 9.66)] compared to those with a negative result [5.41 μg/24 h (IQR: 2.98)], with a *p*-value < 0.001 (Figure 2). Similarly, plasma aldosterone concentrations were higher in the confirmatory-positive group [median 40.4 ng/dL vs. 19.1 ng/dL, *p* = 0.001], while plasma renin activity (PRA) remained comparably suppressed across both groups [0.29 mg/L/h vs. 0.47 mg/L/h, *p* = 0.089]. Hypokalemia was observed exclusively in patients with positive confirmatory test results (10/18, 55.5%), whereas none of the patients with negative tests had hypokalemia. Systolic and diastolic blood pressure values were also significantly elevated in the confirmatory-positive group (systolic BP: 149.5 mmHg vs. 120.5 mmHg, *p* < 0.001; diastolic BP: 97.5 mmHg vs. 81.3 mmHg, *p* < 0.001) (Table 1).

**Figure 3:**
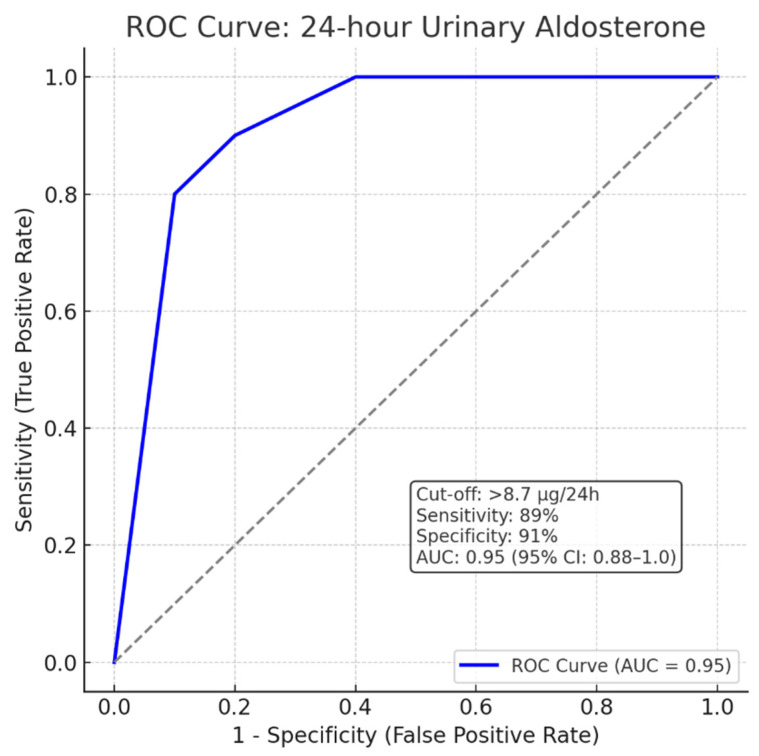
ROC curve for 24 h urinary aldosterone in identifying patients with positive confirmatory tests (*n* = 40). A cut-off >8.7 μg/24 h yielded 89% sensitivity and 91% specificity (AUC: 0.95, 95% CI: 0.881.00).

**Figure 4:**
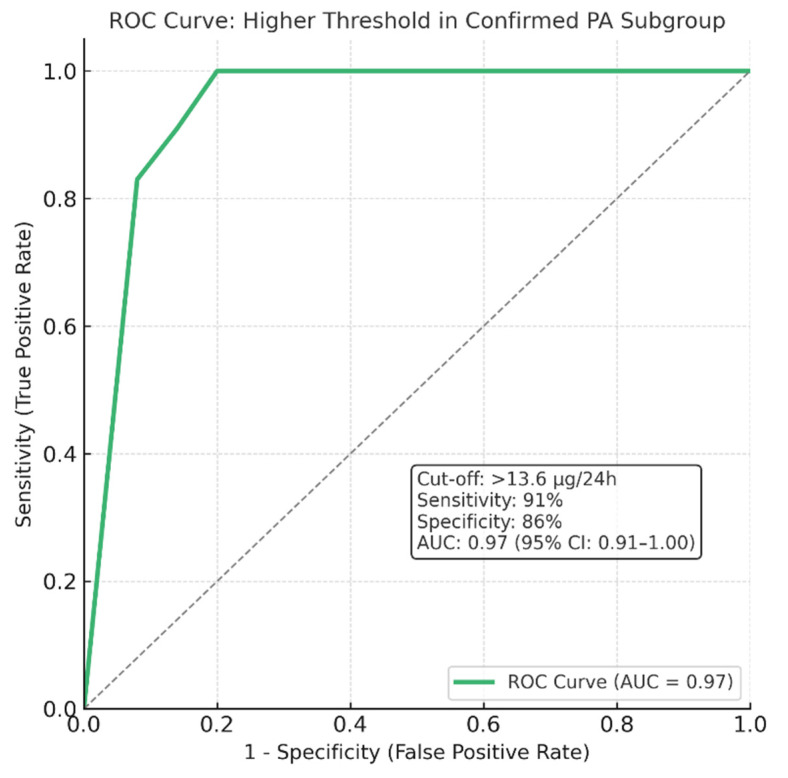
Secondary ROC analysis for 24 h urinary aldosterone in a subgroup of patients with confirmed primary aldosteronism (PA), stratified by post-confirmatory aldosterone levels. A cut-off value of >13.6 μg/24 h demonstrated 91% sensitivity and 86% specificity (AUC: 0.97, 95% CI: 0.91–1.00).

ROC analysis showed that a uAldo cut-off value of >8.7 μg/24 h yielded a sensitivity of 89% (95% CI: 72.2–97.5), specificity of 91% (95% CI: 67.2–96.9), positive predictive value (PPV) of 92.4%, and negative predictive value (NPV) of 87.1%. A higher cut-off of >13.6 μg/24 h demonstrated a sensitivity of 91% (95% CI: 71.8–98.9), specificity of 86% (95% CI: 57.1–98.2), PPV of 88.8%, and NPV of 88.7%. The area under the curve (AUC) for >8.7 μg/24 h was 0.95 (95% CI: 0.88–1.00), and for >13.6 μg/24 h was 0.97 (95% CI: 0.91–1.00) (Table 2, Figure 3 and Figure 4).

According to these cut-offs, patients were stratified into three groups based on uAldo levels: <8.7 μg/24 h (Group 1), 8.7–13.6 μg/24 h (Group 2), and >13.6 μg/24 h (Group 3). Patients in Group 3, with the highest uAldo excretion, had significantly higher systolic and diastolic blood pressure values and lower serum potassium levels. Moreover, plasma aldosterone concentration was markedly elevated in Group 3 (*p* = 0.007), while PRA remained suppressed across all groups (Table 3). These findings support a progressive clinical and biochemical severity profile with increasing uAldo levels. Adrenal imaging findings also differed across groups. Patients in Groups 1 and 2 demonstrated heterogeneous imaging patterns, including normal adrenal imaging, unilateral adrenal adenomas, and bilateral adrenal thickening, whereas all patients in Group 3 had unilateral adrenal adenomas on imaging. Patients in Groups 1 and 2 were managed conservatively with medical treatment and clinical follow-up based on biochemical findings, imaging results, and multidisciplinary evaluation, and none required AVS or surgical intervention during follow-up. This observation suggests that patients with lower or moderate uAldo values and suppressed renin may have a lower likelihood of requiring invasive diagnostic evaluation. Five patients in group 3 underwent AVS, and six patients underwent adrenalectomy. PA was confirmed histologically in all surgical specimens.

## 4. Discussion

In this study, we evaluated the diagnostic value of 24 h urinary aldosterone measurements in patients with suspected PA and compared their performance with classical confirmatory tests. International consensus guidelines recommend the use of uAldo following an oral sodium load as a diagnostic method for patients with suspected PA [30]. This test generally requires a daily sodium intake of 6 g for three consecutive days to achieve urinary sodium excretion exceeding 200 mmol/24 h. However, intravenous or oral sodium loading may pose cardiovascular risks, particularly in individuals with advanced hypertension, heart failure, or renal impairment [10]. Therefore, our study aimed to evaluate the diagnostic performance of 24 h uAldo in patients with suppressed renin activity independent of sodium loading. Notably, there is currently no widely accepted diagnostic cut-off for 24 h uAldo in the Turkish population. While immunoassays were traditionally used to measure urinary aldosterone levels [31], recent studies have indicated that they tend to overestimate concentrations compared to more precise techniques like liquid chromatography–tandem mass spectrometry (LC-MS/MS) [32,33]. In this study, we utilized a fully automated chemiluminescence-based immunoassay for uAldo quantification.

Our results demonstrated that uAldo levels were significantly higher in patients with positive confirmatory test results. A uAldo cut-off value of >8.7 μg/24 h yielded a sensitivity of 89% and a specificity of 91%, while a higher cut-off of >13.6 μg/24 h maintained strong diagnostic performance with 91% sensitivity and 86% specificity. The similar diagnostic performance observed at both thresholds likely reflects the distribution characteristics of our cohort and should be interpreted cautiously given the limited sample size. Larger-scale studies are warranted to refine threshold accuracy and validate the generalizability of our findings. It is noteworthy that our lower uAldo cut-off (>8.7 μg/24 h) is slightly below the conventional diagnostic threshold (10–12 μg/24 h) defined in guidelines for oral sodium loading tests, where adequate sodium excretion (>200 mmol/24 h) is confirmed. This difference should be interpreted cautiously. Because 24 h urinary sodium measurements were not available, actual sodium intake during urine collection could not be objectively verified. Therefore, the observed cut-off may reflect differences in dietary sodium intake, assay methodology, patient selection, and the real-world collection protocol used in our center. Accordingly, the proposed 8.7 μg/24 h threshold should be considered assay- and protocol-specific rather than a direct equivalent of thresholds derived from formal sodium-loading protocols. However, all patients were instructed to maintain a regular sodium diet for at least three days before collection, thereby minimizing extreme variability. Therefore, the proposed 8.7 μg/24 h threshold should be interpreted as a pragmatic cut-off applicable in real-world outpatient settings, rather than as a direct equivalent of the classical ≥12 μg/24 h threshold obtained after sodium loading. Notably, the >13.6 μg/24 h cut-off was derived from a secondary, hypothesis-generating exploratory ROC analysis performed within a subgroup of patients with confirmed PA, who were further stratified by post-confirmatory PAC levels. This enriched subset likely contributed to the high diagnostic performance observed at this threshold. In this context, the >13.6 μg/24 h cut-off may be more appropriately considered as a rule-in or stratification marker for identifying patients who are candidates for adrenal venous sampling (AVS) or surgery, rather than as a general diagnostic threshold for all patients with suspected PA. This interpretation is supported by external evidence, particularly the study by Laney et al., which proposed a similar high-probability uAldo threshold (>49 nmol/24 h, ≈17.6 μg/24 h) as strongly predictive of true PA [30]. Taken together, these findings suggest that the higher threshold identified in our study is consistent with existing data yet remains exploratory and warrants validation in larger multicenter cohorts.

Our findings suggest that certain uAldo thresholds, particularly when interpreted alongside suppressed renin activity, may provide useful complementary information for the evaluation and stratification of patients with suspected PA. All patients included in this study exhibited suppressed renin activity, which minimized the likelihood of false-positive ARR results and reinforced the reliability of uAldo interpretation within a renin-independent context. This is particularly relevant given the emerging evidence that the ARR may miss a substantial proportion of patients with renin-independent aldosterone excess. Brown et al. demonstrated that, in a large cohort including normotensive and hypertensive individuals, the prevalence of biochemical PA was 11.3% and 22.0%, respectively, and many of these cases were not identified using ARR alone. This underscores the potential of uAldo to capture cases across a broader spectrum, including subclinical forms [6]. Urinary aldosterone analysis more accurately reflects the mean aldosterone level than random plasma samples due to the diurnal fluctuations and intra-individual variability in plasma aldosterone levels [14,22]. Similarly, Abdelhamid et al. highlighted the potential of urinary aldosterone metabolites as a reliable screening test for PA [23].

Patients were stratified into three groups based on uAldo levels derived from ROC curve analyses. A lower cut-off of >8.7 μg/24 h, based on the entire cohort, effectively differentiated patients with positive versus negative confirmatory tests. A higher cut-off of >13.6 μg/24 h was obtained from a secondary ROC analysis in a subset of patients with confirmed PA, further stratified by post-confirmatory plasma aldosterone levels (5–10 vs. >10 ng/dL). This dual-threshold approach enabled a risk-adjusted clinical interpretation: patients with uAldo <8.7 μg/24 h, most of whom had negative confirmatory tests, had a low likelihood of PA; those with intermediate levels (8.7–13.6 μg/24 h) were typically managed effectively with medical therapy alone, while those exceeding 13.6 μg/24 h often required further radiological or surgical intervention and were found to have unilateral adenoma. Thus, these thresholds aim to define practical cut-offs that support both diagnosis and individualized clinical decision-making. This two-tiered ROC approach, combining analyses from both the full cohort and the PA-confirmed subset, strengthened the interpretative models. This aligns with findings by Monticone et al., who reported that confirmatory tests may be unnecessary in patients with moderate biochemical findings and high pre-test probability [11]. Patients with uAldo levels >13.6 μg/24 h may be considered for radiological imaging and, if appropriate, invasive procedures such as AVS. Similarly, Wu et al. found that a single 24 h uAldo collection, performed after potassium repletion and prior to saline infusion, demonstrated strong diagnostic performance, comparable even to spot urine aldosterone-to-creatinine ratios [29]. The concordance of our high AUC values (0.95 and 0.97 for the >8.7 and >13.6 μg/24 h thresholds, respectively) with these larger studies reinforces the clinical utility of uAldo as a diagnostic marker.

Taken together, these findings suggest that uAldo may serve not only as a diagnostic tool but also as a stratification marker in clinical management. This stratified approach could enhance diagnostic efficiency and may be particularly valuable in resource-limited settings, potentially reducing the need for unnecessary invasive or resource-intensive testing in selected patients. While our study provides preliminary support for this approach, we acknowledge the modest sample size and have therefore transparently reported 95% confidence intervals for all diagnostic performance metrics. As expected, the intervals are relatively wide, reinforcing the need for future large-scale validation to confirm the generalizability of our proposed cut-offs.

A further strength of our study is the multidisciplinary team approach used in clinical decision-making. Final management decisions regarding further testing, medical therapy, or surgery were made jointly by endocrinologists, radiologists, and surgeons. This real-world approach enhances the clinical applicability of our findings and aligns with current practice guidelines [2,4,12].

Nevertheless, several limitations should be acknowledged. Despite increasing the sample size to 40 patients, it remains modest and comes from a single center, limiting generalizability. Moreover, because our cohort primarily included patients with a high pre-test probability of PA, most of whom were hypertensive and frequently hypokalemic, the diagnostic performance estimates may not fully represent broader or lower-risk screening populations. In addition, subgroup analyses should be interpreted cautiously due to the limited sample size and should be considered exploratory and hypothesis-generating rather than definitive. This may introduce a degree of spectrum bias, and our proposed cut-offs (8.7 and 13.6 μg/24 h) should therefore be validated in larger and more heterogeneous cohorts, including patients with milder or normokalemic phenotypes, to confirm their generalizability. Future multicenter, prospective studies with larger and more diverse populations are warranted to confirm these cut-offs and formally integrate uAldo into diagnostic algorithms for PA.

Although the study involved retrospective analysis, the data were prospectively collected according to a standardized diagnostic protocol, thereby minimizing potential selection and measurement bias. Only patients who successfully completed 24 h urine collection and confirmatory testing were included, and data were retrieved according to a standardized protocol to minimize bias. Nevertheless, as an observational study, unmeasured confounders may exist, and results should be interpreted within the context of our center’s diagnostic workflow.

Dietary sodium intake and medication use were standardized to some extent; full control was not possible. The absence of 24 h urinary sodium (uNa) measurements is therefore a limitation of our study. This limitation is particularly important when interpreting the proposed uAldo cut-offs, since sodium intake directly influences aldosterone secretion and urinary aldosterone excretion. However, our primary aim was to evaluate 24 h urinary aldosterone as a practical and accessible diagnostic tool under real-world conditions rather than within a formal sodium loading protocol. Patients were advised to maintain a regular sodium diet; adherence was assessed through dietary history, and none were using diuretics or following a low-salt regimen during urine collection. Future prospective studies incorporating uNa measurements would further strengthen the interpretation of uAldo results.

In our study, urinary aldosterone levels were measured using a chemiluminescence immunoassay (CLIA). CLIA offers advantages such as automation, rapid turnaround, and broad availability, making it suitable for routine clinical use. However, CLIA can overestimate aldosterone concentrations due to cross-reactivity with aldosterone metabolites, particularly in patients with impaired renal function [34]. In contrast, liquid chromatography-tandem mass spectrometry (LC-MS/MS) provides higher specificity and accuracy by directly quantifying aldosterone, thereby minimizing interference from metabolites [35]. Despite its precision, LC-MS/MS is less accessible due to higher costs and the need for specialized equipment and expertise. Therefore, while CLIA is practical for widespread screening, clinicians should be aware of its limitations. This methodological variability underscores the need to interpret cut-off values in the context of the assay used and to consider standardization across platforms in future research. Importantly, the proposed uAldo cut-offs are assay-dependent. Since chemiluminescence immunoassays may yield slightly higher aldosterone values due to cross-reactivity, the corresponding thresholds could differ when measured by more specific methods such as LC-MS/MS. Nevertheless, our findings are qualitatively consistent with recent data obtained using LC-MS/MS as the reference standard, supporting the robustness of our results across analytical platforms. Although our laboratory did not perform direct calibration against LC-MS/MS, clinicians should be aware that urinary aldosterone thresholds are inherently assay-dependent, and cut-offs may require adjustment based on individual laboratory calibration. There is a growing consensus in the endocrinology community that assay standardization and cross-platform harmonization are essential for reliable interpretation of aldosterone measurements across centers.

Additionally, the relatively high AUC values observed in the ROC analysis (>0.95) should be interpreted with caution. Given the small sample size (*n* = 40), there is a potential risk of overfitting, which may lead to an overestimation of diagnostic performance. Although the post hoc power analysis demonstrated excellent statistical power (100%) for detecting the large observed difference in median uAldo levels between groups (Cohen’s d = 2.13), the study was not designed to detect smaller yet clinically meaningful differences, particularly within subgroups or borderline cases. Therefore, while our findings support the discriminatory capacity of uAldo, the negative predictive value of low uAldo levels (<8.7 μg/24 h) in excluding PA should be interpreted cautiously until validated in larger cohorts. Future multicenter studies with prospective power calculations are necessary to confirm these thresholds and to refine their clinical applicability across diverse patient populations. An independent validation cohort was not available for this study, and thus the proposed thresholds require external validation before broader implementation.

Beyond its diagnostic performance, the practical applicability of 24 h urinary aldosterone testing represents a major advantage. In many clinical settings, performing confirmatory suppression tests or adrenal venous sampling may be logistically challenging or contraindicated. The ability to stratify patients based on urinary aldosterone thresholds could therefore reduce unnecessary invasive testing and help prioritize cases requiring further work-up. This approach aligns with current movements toward simplified, patient-centered, and cost-effective pathways for diagnosing PA.

Blinding was not feasible within our real-world diagnostic workflow, and clinicians were aware of 24 h urinary aldosterone results when making management decisions. This procedure may have introduced incorporation bias. Adrenal venous sampling (AVS) was not performed in Groups 1 and 2, so it is unknown whether some patients in these groups might have harbored unilateral PA that was managed medically. However, their clinical outcomes were acceptable under medical therapy, supporting the safety of our stratification approach. This methodology reflects a pragmatic decision to avoid invasive testing when urinary aldosterone suggested only mild or borderline autonomous aldosterone production. Despite these limitations, the high diagnostic accuracy seen in ROC analysis and consistency with existing literature lend strength to our conclusions.

## 5. Conclusions

The 24 h urinary aldosterone measurement, particularly when interpreted alongside suppressed renin activity, demonstrated promising diagnostic performance in this cohort and may represent a practical and non-invasive adjunctive tool for the evaluation of primary aldosteronism. In patients with a positive screening test and suppressed renin, higher 24 h urinary aldosterone levels were associated with a greater likelihood of primary aldosteronism and may help support clinical stratification and decision-making regarding further evaluation. Conversely, lower uAldo values were associated with a lower probability of clinically significant autonomous aldosterone secretion. However, because confirmatory testing served as the reference standard in this study, these findings should be considered hypothesis-generating and should not be interpreted as evidence that uAldo can replace established confirmatory tests. Larger prospective multicenter studies are required to externally validate the proposed cut-offs and determine the potential role of uAldo within future diagnostic algorithms for primary aldosteronism.

## Figures and Tables

**Table 1:** Demographic, clinical and laboratory findings according to confirmatory test results.

	Confirmatory Test Positive Group (*n* = 18)	Confirmatory Test Negative Group (*n* = 22)	*p*
Age (years) *	49.94 ± 12	53.86 ± 10.68	0.251
Sex (*n*, %)			
Female	7 (38.9)	12 (54.6)	0.708
Male	11 (61.1)	10 (45.4)	
Systolic blood pressure (mmHg)	149.5 ± 11.6	120.5 ± 7.4	**<0.001**
Diastolic blood pressure (mmHg)	97.5 ± 4.3	81.3 ± 4.5	**<0.001**
ALT (U/L)	22.5 ± 8.7	26.64 ± 12.5	0.318
AST (U/L)	17.33 ± 4.6	21.41 ± 8.9	0.18
Urea (mg/dL)	26.67 ± 7.18	30.73 ± 5.78	0.045
Creatinine (mg/dL)	0.83 ± 0.17	0.81 ± 0.16	0.677
Sodium (mEq/L)	141.5 ± 2.6	140.8 ± 2.1	0.443
Potassium (mEq/L)	3.4 ± 0.6	4.3 ± 0.33	**<0.001**
Plasma aldosterone (ng/dL)	40.4 (26.2)	19.1 (11.2)	**0.001**
Plasma renin activity (mg/L/h)	0.29 (0.07)	0.47 (0.65)	0.089
24 h urine aldosterone (µg/24 h)	16.3 (9.66)	5.41 (2.98)	**<0.001**

* Data are presented as median (interquartile range) for non-normally distributed variables and mean ± standard deviation for normally distributed variables. Bold values indicate statistically significant results (*p* < 0.05).

**Table 2:** Diagnostic performance of 24 h urinary aldosterone levels in predicting primary aldosteronism.

Cut-Off Value (μg/24 h)	Sensitivity (%)	Specificity (%)	PPV (%)	NPV (%)	AUC (95% CI)
>8.7	89	91	92.4	87.1	0.95 (0.880.99)
>13.6	91	86	88.8	88.7	0.97 (0.91–1.00)

ROC analysis was used to identify optimal cut-off values for 24 h urinary aldosterone (uAldo) in diagnosing PA. Sensitivity and specificity were calculated using binary classification based on confirmatory test results. Abbreviations: PPV, positive predictive value; NPV, negative predictive value; AUC, area under the curve; CI, confidence interval.

**Table 3:** Demographic, clinical and laboratory findings according to 24 h urinary aldosterone stratification.

	Group 1 (*n* = 21)	Group 2 (*n* = 7)	Group 3 (*n* = 12)	*p*
Age (years) *	53.81 ± 11.83	48.14 ± 8.71	51.42 (11.9)	0.574
Sex (*n*, female/male)	13/8	2/5	4/8	0.156
Systolic blood pressure (mmHg)	122.5 ± 7.9	136.3 ± 7.2	155.5 ± 14.4	**<0.001**
Diastolic blood pressure (mmHg)	82.8 ± 4.6	91.7 ± 4.7	99.3 ± 4.1	**<0.001**
ALT (U/L)	28 ± 12.8	21.29 ± 5.3	21.08 ± 8.9	0.256
AST (U/L)	21.8 ± 8.8	17.6 ± 4.2	16.92 ± 5.23	0.205
Urea (mg/dL)	29.7 ± 5.7	29.4 ± 9	27.2 ± 7	0.513
Creatinine (mg/dL)	0.79 ± 0.15	0.8 ± 0.13	0.88 ± 0.19	0.289
Sodium (mEq/L)	141 ± 2.1	140.7 ± 2.9	141.6 ± 2.5	0.828
Potassium (mEq/L)	4.2 ± 0.3	4.1 ± 0.3	3.1 ± 0.5	**<0.001**
Plasma aldosterone (ng/dL)	17.3 (12.6)	25.9 (14.9)	39.5 (35.7)	**0.007**
Plasma renin activity (mg/L/h)	0.35 (0.61)	0.2 (0.02)	0.2 (0.12)	0.14
24 h urine aldosterone (µg/24 h)	4.95 (2.32)	9.27 (0.52)	18.36 (10.15)	**<0.001**

* Data are presented as median (interquartile range) for non-normally distributed variables and mean ± standard deviation for normally distributed variables. Bold values indicate statistically significant results (*p* < 0.05).

## Data Availability

The datasets presented in this article are not readily available due to ethical and privacy restrictions related to patient data. Requests to access the datasets should be directed to the corresponding author.
